# Identification of candidate biomarkers associated with apoptosis in melanosis coli: GNG5, LPAR3, MAPK8, and PSMC6

**DOI:** 10.1042/BSR20181369

**Published:** 2019-01-18

**Authors:** Xiaohang Hua, Jiangang Chen, Lingli Wu

**Affiliations:** Clinical Laboratory, Hangzhou Cancer Hospital, Hangzhou 310000, Zhejiang, China

**Keywords:** biomarkers, cell apoptosis, differently express, Melanosis coli, protein-protein interaction network

## Abstract

**Purpose:** Melanosis coli (MC) is a disorder of pigmentation of the wall of the colon, often identified at the time of colonoscopy. The aim of the present study is to identify candidate biomarkers for MC. **Methods:** The transcriptome data for MC (GSE78933) with five MC tissues and five corresponding normal tissues is obtained from the NCBI Gene Expression Omnibus (GEO) database. R/Bioconductor package limma was used to screen differently expressed genes (DEGs). ClueGO of cytoscape was applied for Gene Ontology (GO) functional and Kyoto Encyclopedia of Genes and Genomes (KEGG) pathway enrichment. Based on STRING V10 database, protein–protein interaction (PPI) network was constructed. The pathological tissue and normal tissue from 23 MC patients and 23 controls were collected, respectively. The relative expression of hub nodes was detected by qRT-PCR and Western blot. For regulating the expression of these genes, overexpression vector was constructed or siRNA transfection was used. Finally, apoptosis was detected by flow cytometry. **Results:** Total 1342 DEGs were screened, including 786 up-regulated and 556 down-regulated genes. These genes were mainly enriched in stimulatory C-type lectin receptor signaling pathway, polysaccharide biosynthetic process, intracellular, and oxidative phosphorylation. PPI network was then constructed with 426 DEGs and 895 interactions. Thereinto, G-protein subunit γ 5 (GNG5), lysophosphatidic acid receptor 3 (LPAR3), mitogen-activated protein kinase 8 (MAPK8), NHP2L1, proteasome 26S subunit, ATPase 6 (PSMC6), and phosphatidylinositol-4,5-bisphosphate 3-kinase catalytic subunit β (PIK3CB) were hub nodes with higher degree. RT-PCR and Western blot results showed that GNG5, LPAR3, MAPK8, and PSMC6 were differently expressed with significance. The expression of these screened genes is also related with cell apoptosis. **Conclusion:** GNG5, LPAR3, MAPK8, and PSMC6 might be candidate biomarkers associated with apoptosis in MC.

## Introduction

Melanosis coli (MC) is a non-inflammatory bowel disease characterized by melanin deposition in the colonic mucosa [1]. The essence of this disease is that macrophages in the lamina propria of the colonic mucosa contain a large amount of lipofuscin [2]. The incidence rate in males is higher than that of females, and the age of onset is always more than 60 years [3]. The main symptoms are bloating, constipation, and difficulty in defecation, while few patients have symptoms of lower abdominal pain and poor appetite [4]. Some patients are with hypokalemia, hyponatremia, and low blood calcium. There is currently no specific drug treatment for MC. Most scholars believe that MC is a benign and reversible non-inflammatory intestinal mucosal lesion [3]. With the improvement of constipation symptoms and the withdrawal of laxatives, a large amount of lipofuscin is digested and decomposed by lysosomes, and the pigmentation of MC can be alleviated and even disappeared [5]. However, what is frustrating that more patients were with complications such as colon cancer, adenoma, and polyps [6]. Thereby, early detection and treatment of MC are very necessary to reduce the risk of disease and its complications.

In recent years, biological analysis and molecular mechanism analysis are widely used for various diseases. For instance, Liu et al. [7] conducted an analysis on amino acid and cDNA sequence, and confirmed that metallopanstimulin-1 was closely associated with tumorigenesis. Interestingly, the expression of several colorectal carcinoma genes, including *Bcl-2, Ki-67, K-ras*, and *Cox-2*, were related to cells’ mucosal apoptosis in elderly MC patients [8]. Though these mechanisms and genes were researched, complications’ prevention of MC by genetic means has not yet reached. More precise biomarkers need to be studied to promote the progress of this event.

With the implementation of the Human Genome Project, the development of bioinformatics has been greatly promoted, and the ensuing and analysis of the large amount of accounting and protein data has become the main task of biology [9]. However, the results of bioinformatics analysis are often extensive and inaccurate. In order to provide a theoretical basis for researching MC development and preventing complications in the early stages, bioinformatics analysis was used to screen candidate biomarkers of MC, and clinical data and *in vitro* experiments were combined to research the molecular mechanism of screened genes in MC.

## Materials and methods

### Acquisition and preprocessing of data

The expression profile of MC was acquired from Gene Expression Omnibus (GEO, https://www.ncbi.nlm.nih.gov/geo) database of NCBI (GEO accession: GSE78933) with the platform of [HTA-2_0] Affymetrix Human Transcriptome Array 2.0 [transcript (gene) version]. The profile included five colonic mucosa samples in MC groups and five colonic mucosa samples in MC groups. The raw data of expression profile were preprocessed by background correction and standardization. According to the annotation information of the chip, the probes were mapped to the corresponding genes, and the average value was calculated as expression value of each gene.

### Screening of differently expressed genes and enrichment analysis

The moderated T-test of R/Bioconductor package limma was used to screen differently expressed genes (DEGs) between MC and control samples with the threshold of *P*<0.05.

ClueGO is a Cytoscape plug-in that visualizes the non-redundant biological terms for large clusters of genes in a functionally grouped network. It can be also combined with GOlorize. ClueGO charts showed underlying specificity and the common aspects of biological role. The significance of the terms is automatically calculated. ClueGO is easily updatable based on the newest files, including Gene Ontology (GO), Kyoto Encyclopedia of Genes and Genomes (KEGG), and Reactome. In the present study, it was used for GO and KEGG signal enrichment analysis. *P*<0.05 was chosen as the threshold. R/Bioconductor package pathview was used for visualization of significant KEGG signaling pathway.

### Construction of protein–protein interaction network

The interaction between DEGs was extracted from the STRING V10 (https://string-db.org/) database for protein–protein interaction (PPI) construction. The combined score for each protein interaction pair could be obtained from STRINE database, which is distributed from 0 to 1. DEG.PPI was constructed with the threshold of *P*<0.7.

### qRT-PCR detected the expression of hub nodes in DEG.PPI

In order to verify the clinical value of screened hub nodes in DEG.PPI, qRT-PCR was used to detect the expression of hub nodes. First, the pathological tissue and normal tissue from 23 MC patients and 23 controls were collected, respectively. All specimens were collected from colonoscopy of patients in Hangzhou Cancer Hospital from December 2016 to December 2017. The study protocol conformed to the ethical guidelines of the 1975 Declaration of Helsinki, and was approved by Hangzhou Cancer Hospital. All patients signed an informed consent form. The obtained fresh specimens were washed with RNAase-free water, put into RNA later immediately and frozen in −80°C until use. The relative expression of hub nodes was detected by qRT-PCR. Total RNA was extracted by cDNA synthesis kit according to the manufacturer’s instructions (Agilent Technologies, CA, U.S.A.). qRT-PCR was undertaken using SYBR Green Mastermix (Applied Biosystems, CA, U.S.A.). The experiment was repeated for three times. Primers were shown as follows:

GAPDH: (F) 5′-TCG ACA GTC AGC CGC ATC TTC TTT-3′; (R) 5′-ACC AAA TCC GTT GAC TCC GAC CTT-3′;

G-protein subunit γ 5 (GNG5): (F) 5′-AGC ACA GAA CCG GAA ACT TAG-3′; (R) 5′-TCA CTT TTA CGC GGT TGA GTC-3′;

Lysophosphatidic acid receptor 3 (LPAR3): (F) 5′-AGG GCT CCC ATG AAG CTA AT-3′; (R) 5′-TTC ATG ACG GAG TTG AGC AG-3′;

Mitogen-activated protein kinase 8 (MAPK8): (F) 5′-TCT CCA GCA CCC ATA CAT CA-3′; (R) 5′-CCT CCA AAT CC ATTA CCT CC-3′;

NHP2L1: (F) 5′- CAG CTG ACC AAC CAG TTG AA-3′; (R) 5′-AAA TCG TCG CAG ATT GCT TT-3′;

Proteasome 26S subunit, ATPase 6 (PSMC6): (F) 5′-GCT GCG TCC AGG AAG ATT AG-3′; (R) 5′-TGC GAA CAT ACC TGC TTC AG -3′;

Phosphatidylinositol-4,5-bisphosphate 3-kinase catalytic subunit β ( (PIK3CB): (F) 5′-CAT TGA AGT TGT GAG CAC CTC TGA A-3′; (R) 5′-ATG GCT CGG TCC AGG TCA TC-3′.

### Cell culture

Normal colon cell line (FHC) was purchased from ATCC and cultured in DMEM with 10% FBS. The cells were incubated in humidified environment with 5% CO_2_ at 37°C.

### Overexpression or silencing of interest genes

Total RNA was extracted by TRIzol method, and cDNA was synthesized by reverse transcription. According to the mRNA sequence of *GNG5, LPAR3*, and *MSMC6* genes, primers were designed by software Clone Manager 7, PCR was used to amplify the target gene, and product was electrophoresed on 1% agarose gel. The electrophoresis was carried out by QIA-quick’s agarose gel electrophoresis recovery kit. The PCR product and pcDNA3.1 Vector were digested with BamH1 and EcoR1, and the recombinant plasmid was extracted by plasmid extraction reagent. The recombinant plasmid was digested by enzyme and identified. The positive recombinants were identified and transfected with Lipofectamine 2000.

SiRNA (SI02757209) for MAPK8 was synthesized by the Qiagen. The FHC cells were seeded in 12-well plates, and then incubated for more than 12 h in antibiotic-free medium. The siRNA were transfected into FHC cells with the help of Lipofectamine 2000 reagent, and the transfected cells were incubated for 60 h.

### Western blot

Cells were harvested and lysed. Antibodies used for Western blot analysis were GNG5, LPAR3, MAPK8, NHP2L1, PSMC6, and PIK3CB (1:1000, Santa Cruz Biotechnology) and GAPDH 1:3000 (Santa Cruz Biotechnology) were used as loading controls. Signals were visualized using ECL chemiluminescence. Changes in protein expression were quantitated by ImageJ software.

### Flow cytometry

Flow cytometric analysis was processed to detect the apoptosis rate of FHC cells after overexpression or silencing of interest genes. After treatment, FHC cells were stained with 7-aminoactinomycin D and annexin V based on instructions (BD Biosciences, CA, U.S.A.). GACSCalibur flow cytometer (BD Biosciences) was used to analyze the stained cells.

### Statistical analysis

Statistical analysis was performed using SPSS 19.0 software. *t* test was used to compare the mean between two groups. *P*<0.05 was regarded as significantly different.

## Results

### DEGs screening and enrichment analysis

Compared with controls, a total of 1342 DEGs, including 786 up-regulated and 556 down-regulated were screened. These screened DEGs were mainly enriched in several GO terms, such as polysaccharide biosynthetic process (*P*=0.007), mitochondrial proton-transporting ATP synthase complex, coupling factor F(o) (*P*=6.10E-11), and innate immune response activating cell surface receptor signaling pathway (*P*=0.014) (Table 1). Simultaneously, these DEGs were involved in a number of pathways, including Oxidative phosphorylation (*P*=0.003), Alanine, aspartate and glutamate metabolism (*P*=0.043) and Glycerolipid metabolism (*P*= 0.045) (Table 2).

**Table 1:** Top six functional terms enriched by DEGs

GO ID	GO term	Nr. genes	Term *P*-value
GO:0000271	Polysaccharide biosynthetic process	10	0.007
GO:0002220	Innate immune response activating cell surface receptor signaling pathway	11	0.048
GO:0000276	Mitochondrial proton-transporting ATP synthase complex, coupling factor F	4	6.10E-11
GO:0000315	Organellar large ribosomal subunit 5	5	0.031
GO:0002220	Innate immune response activating cell surface receptor signaling pathway	11	0.014
GO:0002223	Stimulatory C-type lectin receptor signaling pathway	11	0.012

**Table 2:** KEGG signaling pathway enriched by DEGs

GOID	Ontology source	GO term	Nr. Genes	% Associated genes	Term *P*-value
KEGG:00190	KEGG_09.11.2015	Oxidative phosphorylation	16	12.03008	0.002893328
KEGG:00250	KEGG_09.11.2015	Alanine, aspartate, and glutamate metabolism	5	14.28571	0.043279967
KEGG:00561	KEGG_09.11.2015	Glycerolipid metabolism	7	11.86441	0.044958189
KEGG:03050	KEGG_09.11.2015	Proteasome	10	22.72727	1.18E-04
KEGG:04910	KEGG_09.11.2015	Insulin signaling pathway	14	10.07194	0.022857026
KEGG:05010	KEGG_09.11.2015	Alzheimer’s disease	15	8.928572	0.048119193

### Construction of PPI network

**Figure 1:**
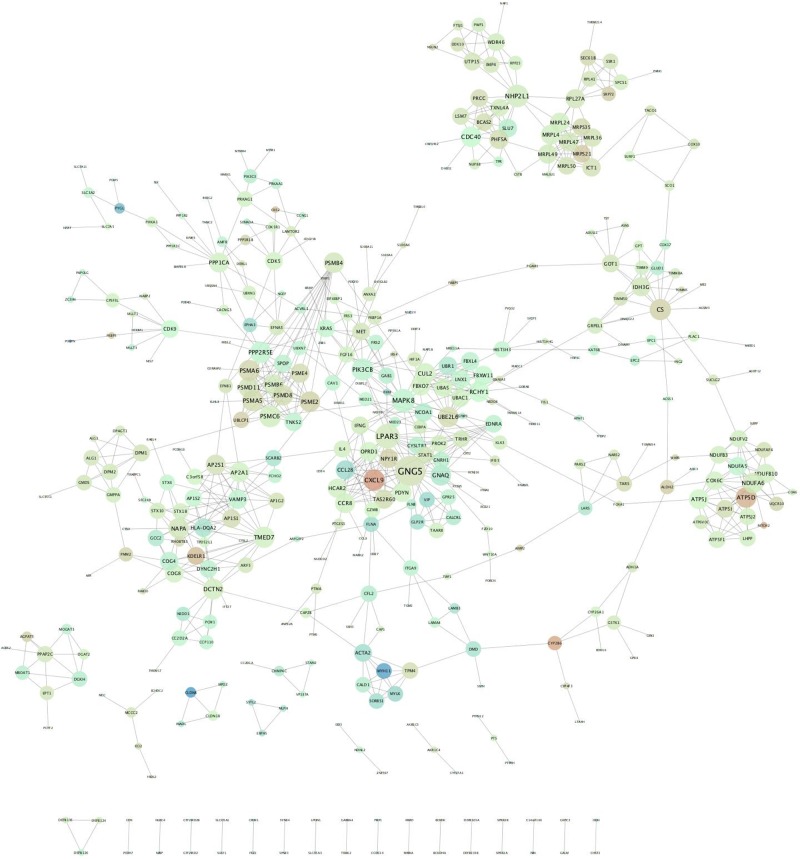
PPI networks The nodes present screened DEGs, while the edges present their relationships. The color of Node from green to red indicates the change in the degree of differential expression logFC from negative to positive. The Node size grows from small to large, indicating a change in the node degree.

DEG.PPI network was successfully constructed with 426 DEGs and 895 edges (Figure 1). The distribution of node degrees in the network approximated the exponential distribution (correlation = 0.896, R-squared = 0.797). Therefore, the network is a scale-free network (SCN). The nodes with higher degrees were regarded as hub genes. Top six genes with the highest degree were shown, including GNG5 (logFC = 0.323), LPAR3 (logFC = 0.246), MAPK8 (logFC = −0.189), NHP2L1 (logFC = 0.213), PSMC6 (logFC = 0.202), and PIK3CB (logFC = 0.212) (Table 3).

**Table 3:** Top six hub nodes of DEG.PPI

Gene	Degree	logFC
*GNG5*	26	0.323217200000002
*LPAR3*	18	0.246190400000001
*MAPK8*	16	−0.1897492
*NHP2L1*	15	0.2130782
*PSMC6*	14	0.202464600000002
*PIK3CB*	14	−0.211626399999998

### The expression level of hub nodes in DEG.PPI

**Figure 2:**
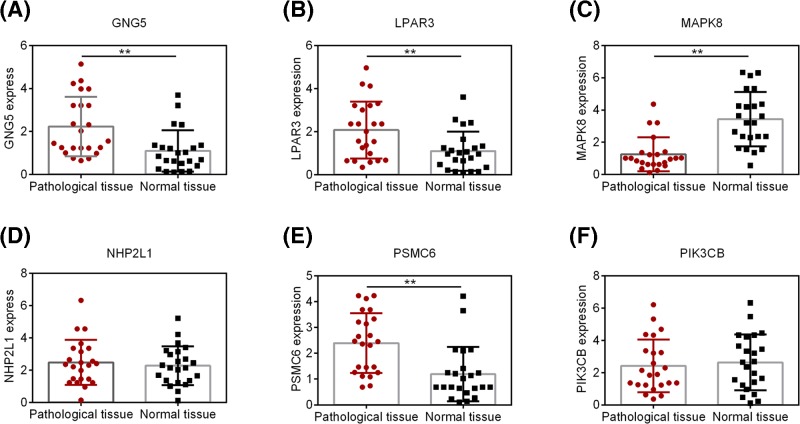
The expression level of hub nodes in pathological tissue and normal tissue (**A**) GNG5; (**B**) LPAR3; (**C**) MAPK8; (**D**) NHP2L1; (**E**) PSMC6; (**F**) PIK3CB.**P<0.01 compared with Normal tissue.

**Figure 3:**
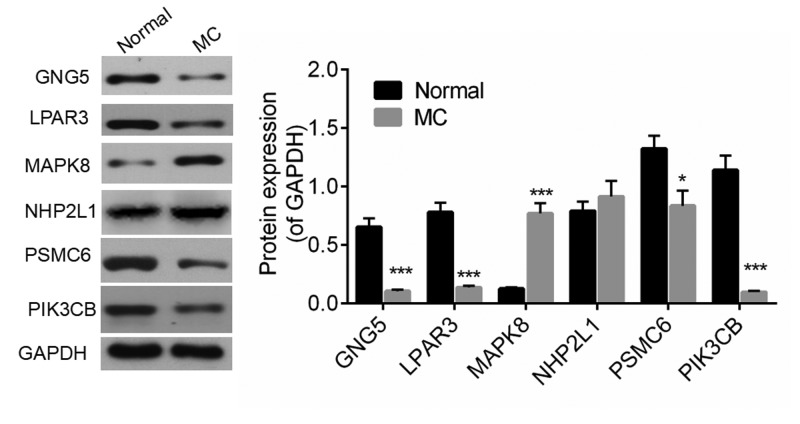
Western blot detected the expression level of hub nodes *P<0.05, ***P<0.001 compared with Normal group.

The expression levels of top six DEGs in DEG.PPI network were detected by qRT-PCR. As shown in Figure 2, the expression levels of GNG5, LPAR3, MAPK8, and PSMC6 were with significant difference between pathological and normal tissue, while no different expression of Small Nuclear Ribonucleoprotein 13 (NHP2L1) and PIK3CB were found between two groups. It is worth noting that consistent with bioinformatics analysis, the expression levels of GNG5, LPAR3, and Proteasome 26S Subunit, ATPase 6 (PSMC6) were up-regulated; the expression level of MAPK8 was down-regulated. Similarly, with the results of qRT-PCR, Western blot assay also confirmed that GNG5, LPAR3, and PSMC6 were significantly overexpressed, while MAPK8 was expressed lower in pathological tissue (Figure 3).

### Cell apoptosis

**Figure 4:**
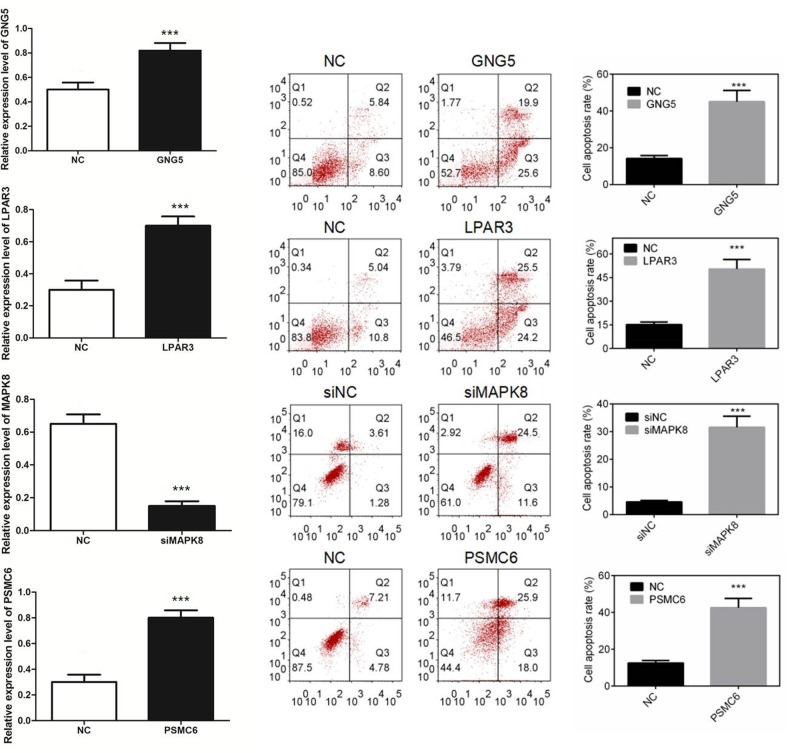
Cell apoptosis of colon cell line FHC by regulating the overexpression of GNG5, LPAR3, PSMC6, or silencing the expression of MAPK8 ***P<0.001 compared with NC group.

By overexpression or silencing of above four genes, Western blot was used to detect if the positive recombinants and siRNA were successfully transfected. As shown in Figure 4, the expression of GNG5, LPAR3, and PSMC6 were significantly up-regulated, while MAPK8 was down-regulated. By up-regulating the expression of GNG5, LPAR3, and PSMC6 or down-regulating the expression of MAPK8, the cell apoptosis rate of FHC cells were significantly higher. The result indicated that the expression levels of GNG5, LPAR3, PSMC6, and MAPK8 were closely related to cell apoptosis (Figure 4).

## Discussion

In order to screen accurate biomarkers and provide a theoretical basis for researching MC development and preventing complications in the early stages, bioinformatics analysis was used to screen candidate biomarkers of MC, and clinical data and *in vitro* experiments were combined to research the molecular mechanism of screened genes in MC. And the results, total six key genes, including GNG5, LPAR3, MAPK8, NHP2L1, PSMC6, and PIK3CB were hub nodes with higher degree in PPI network of MC. RT-PCR and Western blot results confirmed that GNG5, LPAR3, MAPK8, and PSMC6 were differently expressed with significance, and flow cytometry results further showed that expression of these screened genes related with cell apoptosis.

GNG5 encodes a member of G protein, which plays a critical role in internalization, trafficking, and signaling pathways of various G-protein coupled receptors [10]. As shown in previous studies, G-protein could induce apoptotic response in various cancers, such as murine colon adenocarcinoma, human melanoma cells [11,12]. Interestingly, RGS6, a G-protein inactivator, was confirmed to mediate doxorubicin-induced myocardial cell apoptosis [13]. Based on the above information, G-protein and its’ inactivator could regulate cell apoptosis process in cancer. In the present study, GNG5 was screened and involved in functions of generation of precursor metabolites and energy, and contractile fiber. So far, fiber colonoscopy was the main and accurate detection method to diagnose the MC [14]. Thereby, GNG5 might participate in pathogenesis of MC by regulating contractile fiber and apoptotic response.

LPAR3 and PSMC6 were also up-regulated DEGs in MC patients. In the present study, LPAR3 was found to participate in regulation of protein phosphorylation. Moreover, the protein phosphorylation was required for anti-apoptotic function [16]. Importantly, Ali et al. [17] found that serine phosphorylation of vasodilator-stimulated phosphoprotein regulated cell survival and apoptosis of colon cancer. Besides, PSMC6 was involved in innate immune response activating cell surface receptor signaling pathway and pathway of proteasome in this study. In human multiple myeloma cells, lacking the PSMC6 expression might induce resistance to apoptosis [18].

It is worth noting that, MAPK8 was screened and identified as the unique down-regulated gene of MC patients in the present study. In addition, it was involved in regulation of innate immune response and pathway of insulin signaling pathway. Similarly with previous study, phosphorylation of MAPK8 was related with cell apoptosis [15]. A case–control study of Corredoira-Sanchez et al. [19] showed that the relationship between *Streptococcus gallolyticus* subsp. *gallolyticus* and colorectal neoplasia has been established, and innate immune response played an important role in this process. So far, no evidence confirmed the connection between MC and insulin signaling pathway. However, a number of evidence confirmed that the differently expressed MAPK8 or phosphorylation of MAPK8 was associated with cell apoptosis [15]. Thence, MAPK8 might regulate the process of immunity and apoptosis, and further effect the development of MC.

Based on the above evidence, we found that the screened key genes were more or less associated with apoptosis. Previous studies have shown that constipation and long-term oral laxatives are the leading causes of colonic melanosis [20]. Amongst them, oral steroids and diphenylmethane laxatives are the most important drugs. The study of Wang et al. [21] have confirmed that the apoptosis rate of colonic melanosis group was significantly higher than that of the control group. When the various laxatives enter the colon, the drug could cause transient, meter-related apoptosis of intestinal epithelial cells [22]. Consistent with the results of this study, after up-regulating the expression of GNG5, LPAR3, and PSMC6, or down-regulating the expression of MAPK8, the cell apoptosis rate of FHC cells were significantly higher. Thereby, the expression of GNG5, LPAR3, MAPK8, and PSMC6 were closely related with apoptosis of normal colonic mucosal cells.

In conclusion, GNG5, LPAR3, MAPK8, and PSMC6 might be candidate biomarkers associated with apoptosis in MC. The result might provide a theoretical basis for researching MC development and preventing complications in the early stages.

## Highlights

1. Total 1342 DEGs were screened between MC and controls.
2. PPI network with 426 DEGs and 895 edges was constructed.
3. GNG5, LPAR3, MAPK8, and PSMC6 were differently expressed in pathological and normal tissues.
4. GNG5, LPAR3, MAPK8, and PSMC6 were related to cell apoptosis.

## Abbreviations

ATCC: American type culture collection ATCC
DEG: differently expressed gene
DMEM: Dulbecco’s Modified eagle medium
FHC: Fetal human cells
GEO: Gene Expression Omnibus
GNG5: G-protein subunit γ 5
GO: gene ontology
KEGG: Kyoto Encyclopedia of Genes and Genomes
logFC: log Fold change
LPAR3: lysophosphatidic acid receptor 3
MAPK8: mitogen-activated protein kinase 8
MC: melanosis coli
PIK3CB: phosphatidylinositol-4,5-bisphosphate 3-kinase catalytic subunit β
PPI: protein–protein interaction
PSMC6: proteasome 26S subunit, ATPase 6
qRT-PCR: quantitative real-time quantitative polymerase chain reaction
RT-PCR: real-time quantitative polymerase chain reaction
SCN: scale-free network
